# UPLC-Q-TOF-MS/MS Analysis of *Seco*-Sativene Sesquiterpenoids to Detect New and Bioactive Analogues From Plant Pathogen *Bipolaris sorokiniana*

**DOI:** 10.3389/fmicb.2022.807014

**Published:** 2022-03-09

**Authors:** Yan-Duo Wang, Jian Yang, Qi Li, Yuan-Yuan Li, Xiang-Mei Tan, Si-Yang Yao, Shu-Bin Niu, Hui Deng, Lan-Ping Guo, Gang Ding

**Affiliations:** ^1^Key Laboratory of Bioactive Substances and Resources Utilization of Chinese Herbal Medicine, Ministry of Education, Institute of Medicinal Plant Development, Chinese Academy of Medical Sciences and Peking Union Medical College, Beijing, China; ^2^State Key Laboratory Breeding Base of Dao-di Herbs, National Resource Center for Chinese Materia Medica, China Academy of Chinese Medical Sciences, Beijing, China; ^3^Department of Pharmacy, Beijing City University, Beijing, China; ^4^Key Laboratory of Microbial Resources, Ministry of Agriculture and Rural Affairs, Institute of Agricultural Resources and Regional Planning, Chinese Academy of Agricultural Sciences, Beijing, China

**Keywords:** *Bipolaris sorokiniana*, *seco*-sativene sesquiterpenoids, McLafferty rearrangement, NMR analysis, antioxidant activity

## Abstract

*Seco*-sativene sesquiterpenoids are an important member of phytotoxins and plant growth regulators isolated from a narrow spectrum of fungi. In this report, eight *seco*-sativene sesquiterpenoids (**1**–**8**) were first analyzed using the UPLC-Q-TOF-MS/MS technique in positive mode, from which their mass fragmentation pathways were suggested. McLafferty rearrangement, 1,3-rearrangement, and neutral losses were considered to be the main fragmentation patterns for the [M+1]^+^ ions of **1**–**8**. According to the structural features (of different substitutes at C-1, C-2, and C-13) in compounds **1**–**8**, five subtypes (A–E) of *seco*-sativene were suggested, from which subtypes A, B/D, and E possessed the diagnostic daughter ions at *m*/*z* 175, 189, and 203, respectively, whereas subtype C had the characteristic daughter ion at *m*/*z* 187 in the UPLC-Q-TOF-MS/MS profiles. Based on the fragmentation patterns of **1**–**8**, several known compounds (**1**–**8**) and two new analogues (**9** and **10**) were detected in the extract of plant pathogen fungus *Bipolaris sorokiniana* based on UPLC-Q-TOF-MS/MS analysis, of which **1**, **2**, **9**, and **10** were then isolated and elucidated by NMR spectra. The UPLC-Q-TOF-MS/MS spectra of these two new compounds (**9** and **10**) were consistent with the fragmentation mechanisms of **1**–**8**. Compound **1** displayed moderate antioxidant activities with IC_50_ of 0.90 and 1.97 mM for DPPH and ABTS^+^ scavenging capacity, respectively. The results demonstrated that *seco*-sativene sesquiterpenoids with the same subtypes possessed the same diagnostic daughter ions in the UPLC-Q-TOF-MS/MS profiles, which could contribute to structural characterization of *seco*-sativene sesquiterpenoids. Our results also further supported that UPLC-Q-TOF-MS/MS is a powerful and sensitive tool for dereplication and detection of new analogues from crude extracts of different biological origins.

## Introduction

*Seco*-sativenes are a member of sesquiterpenoids possessing a unique bicyclo[3.2.1]octane ring system and different substitutions including glycosylation, methylation, and acylation; different heterocyclic rings such as lactone, furan, and pyran ring; and diverse oxygenation sites (hydroxylation) on the core skeleton, increasing the chemical diversity. The structural differences of *seco*-sativenes mainly lie in the diverse substituents at C-1, C-2, and C-13 (Li et al., 2020a). From the structural features, it is implied that *seco*-sativenes come from a sesquiterpene pathway but not from a direct farnesyl pyrophosphate cyclization product. Rearrangement and oxidative cleavage reactions might play a pivotal role in the biosynthetic pathway, which is supported by the isolation of different precursors and intermediates (Mayo et al., 1962a,^1965^; Mayo and Williams, 1965; Li et al., 2020a,b). Fungus *B. sorokiniana* is known for producing a variety of secondary metabolites, with sesterterpene, cyclic peptides, and sesquiterpenoids as the most representative classes (Nihashi et al., 2002; Ali et al., 2016; Qader et al., 2017; Phan et al., 2019). Qader et al. (2017) isolated three *seco*-sativene sesquiterpenoids from *B. sorokiniana*, in which helminthosporal acid and helminthosporol displayed a strong phytotoxic effect on lettuce seed germination and toxicity against brine shrimps, and helminthosporal acid also showed antifungal activity. Phan et al. (2019) isolated and elucidated 12 *seco*-sativene sesquiterpenoids including a new *seco*-sativene sesquiterpenoid and three new sativene analogues from *B. sorokiniana*, in which helminthosporic acid and dihydroprehelminthosporol displayed weak necrotic activity against wheat leaves and helminthosporol showed an inhibitory effect on seed germination. *Seco*-sativene analogues displayed strong phytotoxic effects on cereals and gramineous plants, (Ludwig et al., 1956; Ludwig, 1957; Mayo et al., 1961, 1962b,^1963^; Spencer, 1965; Katsumi et al., 1967; Taniguchi and White, 1967; White and Taniguchi, 1972; Pena-Rodriguez et al., 1988; Pena-Rodriguez and Chilton, 1989; Qader et al., 2017; Phan et al., 2019) whereas others possessed plant-growth-promoting biological activities to rice, lettuce, cucumber, and wheat seedlings (Briggs, 1966; Hashimoto et al., 1967; Nukina et al., 1975; Pena-Rodriguez and Chilton, 1989; Miyazaki et al., 2017, 2018; Qader et al., 2017). In addition, some *seco*-sativene sesquiterpenoids also possessed antifungal, cytotoxic, and toxic effects, and other analogues could inhibit the growth of the malaria-causing protozoan of *Plasmodium falciparum* and exhibited certain anti-NO production activities (Li et al., 2020b). The novel core skeleton and diverse biological activities attracted us to chemically investigate this unique member of sesquiterpenoids. Recently, a series of new *seco*-sativene sesquiterpenoids were isolated from the endophytic fungus *Cochliobolus sativus* (teleomorph: *Bipolaris sorokiniana*) inhabiting in a desert plant, *Artemisia desertorum*, and their structures were mainly determined by NMR experiments, X-ray diffraction, and high-resolution mass analysis. Helminthosporic acid (**2**) could promote plant leaf growth, whereas cochliobolin F, helminthosporic acid (**2**), drechslerine B (**8**), and helminthosporal acid displayed strong phytotoxic effects on corn leaves (Li et al., 2020a). However, the traditional isolation method was used as the main technique for the isolation of *seco*-sativene sesquiterpenoids, (Ramos et al., 2019) which precluded discovery of new/novel analogues of *seco*-sativene sesquiterpenoids. Thus, efficient approaches for mining novel structures of *seco*-sativene sesquiterpenoids are urgent.

Mass spectrometry, especially tandem mass spectrometry, has been one of the most important physicochemical approaches for the characterization of secondary metabolites due to its rapidity and sensitivity (Jin et al., 2018; Liang et al., 2018; Conceição et al., 2020; Scupinari et al., 2020). Molecular weight and formula are often inconclusive for metabolite identification; however, fragmentation patterns represent a specific feature for a certain structural class. Chen et al. (2018) applied neutral loss scan in QqQ-MS and molecular formula calculation in UPLC-Q-TOF-MS to detect amorfrutin analogues, which provided the idea of detection and structural dereplication in the complex crude extract. Yang et al. (2017) used UPLC-Q-TOF-MS/MS coupled with neutral loss scan and diagnostic ions to analyze the secondary metabolites of *Schisandra chinensis*. Ahad et al. (2020) combined UPLC-Q-TOF-MS with SCX-SPE to achieve the enrichment and structural identification of the same skeleton metabolites. Thus, much evidence demonstrated that fragmentation patterns coupled with UPLC-Q-TOF-MS/MS analysis were an efficient and convenient tool for the detection and dereplication of similar metabolites.

**FIGURE 1:**
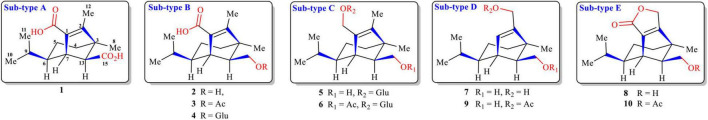
Structures of *seco*-sativene sesquiterpenoids (**1–10**).

A series of *seco*-sativene sesquiterpenoids (**1–8**, Figure 1) were isolated and elucidated in our previous work (Li et al., 2020a). According to the structural features (of different groups at C-1, C-2, and C-13), five subtypes of *seco*-sativenes were suggested (subtypes A-E) (Figure 1). Interestingly, each subtype of the structure has the same diagnostic daughter ions in the mass spectrometric profile, which could provide a reliable approach to analyze structures of *seco*-sativenes and target potent new analogues. To date, no investigations about electrospray tandem mass/mass of *seco*-sativenes sesquiterpenoids were reported. The potential application prospect and unique skeleton of *seco*-sativenes prompted us to investigate the mass spectrometric cleavage mechanisms of this unique member of sesquiterpenoids.

In this report, the UPLC-Q-TOF-MS/MS fragmentation rules of *seco*-sativene sesquiterpenoids (**1–8**) were presented; some known and new *seco*-sativene sesquiterpenoids were detected from the extract of the plant pathogen fungus *Bipolaris sorokiniana* based on UPLC-Q-TOF-MS/MS analysis. Two new (**9** and **10**) and two known (**1** and **2**) *seco*-sativene sesquiterpenoids were then isolated and elucidated by HR-ESI-MS and NMR spectra, and the antioxidant activities of these *seco*-sativene sesquiterpenoids (**1, 2, 9**, and **10**) were assessed.

## Materials and Methods

### General Experimental Procedures

Optical rotations were measured on a 241 polarimeter (PerkinElmer, Waltham, United States). UV-2102 (Unico, Shanghai, China) was used to record UV data. IR spectra were recorded on an FTIR-8400S spectrophotometer (Shimadzu, Kyoto, Japan). NMR data were acquired on a Bruker 500 spectrometer using solvent signal (CDCl_3_; δ_H_ 7.26/δ_C_ 77.6) as reference. Sephadex LH-20 and silica gel were purchased from Pharmacia (Biotech, Sweden) and Shanghai Titan Scientific Co., Ltd. (Shanghai, China), respectively. Semi-preparative HPLC separation was performed on a SEP LC-52 with an MWD UV detector (Separation (Beijing) Technology Co Ltd., Beijing, China) packed with a YMC-Pack ODS-A column. HR-ESI-MS spectra were analyzed using an ESI-Q-TOF-MS (Waters Xevo G2-XS QTof, United States).

### General Experimental Procedures

Eight *seco*-sativene sesquiterpenoids were analyzed using a UPLC-Q-TOF-MS/MS system (Waters, United States). Chromatographic analysis was carried out with a Waters ACQUITY UPLC-PDA system equipped with an analytical reverse-phase C-18 column (2.1 × 100 mm, 1.7 μm, ACQUITY BEH, Waters, United States) with an absorbance range of 200 to 400 nm. The column temperature was maintained at 40°C. As the mobile phase, 0.1% formic acid in water (A) and 0.1% formic acid in acetonitrile (B) were used. The gradient conditions were as follows: 0–2 min, 35% B; 2–17 min, 35–98% B; 17–19 min, 98% B; and 19.1–21 min, 35% B. The flow rate from the UPLC system into the ESI-Q-TOF-MS detector was 0.3 ml/min. The auto-injected volume was 0.3 μl. Time-of-flight MS detection was performed with the Xevo G2-XS QTof system (Waters) combined with an ESI source in positive ion scan mode. The desolvation temperature was set at 450°C with desolvation gas flow at 900 L/h, and the source temperature was 80°C. The lock mass in all analyses was leucine-enkephalin [(M+H)^+^ = 556.2771], used at a concentration of 200 μl/ml and infused at a flow rate of 10 L/min. Raw data were acquired using the centroid mode, and the mass range was set from *m*/*z* 100 to 1,000. The capillary voltage was set at 2.5 kV with 30 V of sample cone voltage. The collision energy was set as 6 eV for low-energy scan and a ramp from 30 to 50 eV for high-energy scan. The instrument was controlled by MassLynx 4.1 software.

### Strain and Fermentation

The strain of *Bipolaris sorokiniana* (strain number: ACCC36805) was isolated from the seed of wheat and provided by the Chinese Academy of Agricultural Sciences. The fungus was grown on PDA (potato dextrose agar) plates at 25°C for 10 days. Then the fresh mycelium was inoculated into the autoclaving sterilized solid medium with the formula of rice (60.0 g) and distilled water (80 ml) in Fernbach flasks (500 ml) for further fermentation at 25°C for 30 days.

### Extraction and Isolation

The fermented rice substrate was extracted with EtOAc three times, and the solvent was evaporated to dryness under vacuum to afford 200 g of crude extract. The original extract was fractionated on a silica gel column using petroleum ether-acetone (1:0–0:1) progressively to give five fractions (Fr. 1 to Fr. 5). Fr. 2 (8.0 g) was separated on a silica gel column to obtain eight fractions (Fr. 2.1 to Fr. 2.8). Fr. 4 (27.8 g) was separated on a silica gel column to obtain five parts (Fr. 4.1 to Fr. 4.5). Fr. 4.1 was separated on a silica gel column and RP-HPLC (0–5 min 80% MeOH in H_2_O, 5–25 min 80–100% MeOH in H_2_O, 5 ml/min) to obtain Fr. 4.1.1 (13.8 mg, *t*_R_ = 10.5 min). Fr. 4.1.1 (13.8 mg) was purified by semi-preparative HPLC (0–5 min 70% acetonitrile in H_2_O, 5–25 min 70–90% acetonitrile in H_2_O, 2 ml/min) to obtain compound **10** (1.8 mg, *t*_R_ = 15.2 min). Fr. 4.2 (5.6 mg) was purified by semi-preparative HPLC (60% acetonitrile in H_2_O, 2.5 ml/min) to obtain compound **9** (2.3 mg, *t*_R_ = 23.5 min). Fr. 4.3 (3.5 g) was purified by semi-preparative HPLC (0–25 min 80–100% MeOH in H_2_O, 5 ml/min) to obtain compound **2** (230.1 mg, *t*_R_ = 10.3 min). Fr. 4.4 (4.02 g) was separated on a silica gel column, Sephadex LH-20 (dichloromethane:methanol = 1:1 v/v) and semi-preparative HPLC to obtain four fractions (Fr. 4.4.1–Fr. 4.4.4). Fr. 4.4.1 (24.7 mg) was purified by semi-preparative HPLC (75% MeOH in H_2_O, 2 ml/min) to obtain **1** (10.0 mg, 21.6 min).

#### 12-Acetyl-Drechslerine A (9)

Colorless oil: ${\left[\alpha \right]}_{\text{25}}^{D}$-18 (c 0.1, MeOH); UV (MeOH) λ_max_ (log ε) 215 (2.98) nm; IR (neat) ν_max_ 3,420, 2,931, 1,742, 1,456, 1,367, 1,235, 1,031 cm^–1^; for ^1^H NMR and ^13^C NMR data, see Table 1; positive HR-ESI-MS: *m*/*z* 267.1957 [calcd. for C_16_H_27_O_3_ [M+H]^+^, 267.1960].

**TABLE 1:** NMR spectroscopic data of compounds **9** and **10** in CDCl_3_.

Pos.	9					10				
	δ_H_*^a^* (*J* in Hz)	δ_C_*^b^*, mult.	^1^H-^1^H COSY	HMBC	NOESY	δ_H_*^a^* (*J* in Hz)	δ_C_*^b^*, mult.	^1^H-^1^H COSY	HMBC	NOESY
1	5.64, s	127.5, CH	H7	C3, C6, C12, C13			134.8, C		H7, H12, H13	
2		141.0, C		H4, H7, H8, H12, H13			177.2, C		H4, H7, H8, H12, H13	
3		47.0, C		H1, H4, H5, H7, H8, H12, H13, H14			46.9, C		H4, H5, H7, H8, H13, H14	
4	1.36, ddd (13.5, 6.5, 1.5)	34.5, CH_2_	H5	C2, C3, C5, C6, C8, C13		1.57, m	34.5, CH_2_	H15	C2, C3, C5, C6, C8, C13	
	1.29, m									
5	1.67, m	25.1, CH_2_	H4, H6	C3, C4, C6		1.89, m	25.8, CH_2_	H4, H6	C3, C4, C6, C7	
	1.11, m					0.89, m				
6	1.09, m	43.9, CH	H7	C4, C7, C10, C11, C13	H13	1.21, m	43.8, CH	H5, H7, H9	C9, C10, C11	H13
7	2.70, s	42.2, CH	H6, H13	C3, C5, C6, C9, C13, C14	H10	3.04, s	41.0, CH	H1, H6, H13	C1, C2, C3, C5, C6, C14	H10
8	1.01, s	18.6, CH_3_		C2, C3, C4, C13		1.16, s	18.1, CH_3_		C2, C3, C4, C13	
9	1.21, dtd (13.5, 6.5, 2.0)	32.5, CH	H6, H10, H11	C5, C6, C10, C11		1.26, m	32.4, CH	H6, H10, H11	C5, C6, C10, C11	
10	0.93, d (6.5)	21.2, CH_3_	H9, H11	C6, C9, C11	H7	1.04, d (6.0)	21.5, CH_3_	H9, H11	C6, C9, C11	H7
11	0.83, d (6.5)	20.8, CH_3_	H9, H10	C6, C9, C10		0.83, d (6.0)	20.7, CH_3_	H9, H10	C6, C9, C10	
12	4.59, d (13.5)	62.9, CH_2_		C1, C2, C3, C2′		4.83, dd (18.0, 1.5)	67.5, CH_2_		C1, C2	
	4.52, d (13.5)					4.73, d (18.0)				
13	1.57, dd (8.5, 5.0)	62.6, CH	H7, H14	C1, C2, C3, C4, C6, C14	H6	2.16, dd (9.0, 5.5)	63.5, CH	H7, H14	C1, C2, C3, C4, C6, C14	H6
14	3.73, dd (10.5, 5.0)	63.0, CH_2_	H13	C3, C7, C13		4.24, dd (11.0, 5.5)	64.0, CH	H13	C3, C7, C13, C2′	
	3.50, dd (10.5, 8.0)					3.82, dd (11.0, 9.0)				
15							170.8, C		H12	
1′	2.07, s	21.0, CH_3_		C12, C2′		2.05, s	21.2, CH_3_		C14, C2′	
2′		171.2, C		H12, H1′			171.2, C		H14, H1′	

*^a^Recorded at 500 MHz.*

*^b^Recorded at 125 MHz.*

#### 14-Acetyl-Drechslerine B (10)

White powder: ${\left[\alpha \right]}_{\text{25}}^{D}$-16 (c 0.1, MeOH); UV (MeOH) λ_max_ (log ε) 205 (2.84), 234 (2.97) nm; IR (neat) ν_max_ 2,958, 1,747, 1,645, 1,456, 1,367, 1,338, 1,234, 1,031 cm^–1^; for ^1^H NMR and ^13^C NMR data, see Table 1; positive HR-ESI-MS: *m*/*z* 293.1683 [calcd. for C_17_H_25_O_4_ [M+H]^+^, 293.1675].

### Antioxidant Activity

#### DPPH Scavenging Capacity

Take 15 μl of compounds **1**, **2**, **9**, and **10** with a concentration of 10 mM/L and a serial dilution of seven times, and then mix with DPPH solution. After 30 min, the remaining amount of the DPPH radical was measured spectrophotometrically at 517 nm. In this test, for comparison, V_C_ was considered as the positive control, and ethanol was considered as the negative control.

The clearance rate *E* is *E* = [1 – (A_S_ – A_0_)/(A_C_ – A_0_)] × 100%, where A_0_ is the absorbance of the water, A_C_ is the absorbance of ethanol solution, and A_S_ is the absorbance after adding the sample solution. The IC_50_ value was processed by GraphPad Prism 8.

#### ABTS^+^ Scavenging Capacity

Take 15 μl of compounds **1**, **2**, **9**, and **10** with a concentration of 10 mM/L and a serial dilution of seven times, and then mix with ABTS^+^ solution. After 6 min, the remaining amount of the ABTS^+^ radical was measured spectrophotometrically at 405 nm. In this test, for comparison V_C_ served as the positive control, and ethanol served as the negative control.

The clearance rate *E* is *E* = [1 – (A_S_ – A_0_)/(A_C_ – A_0_)] × 100%, where A_0_ is the absorbance of the water, A_C_ is the absorbance of ethanol solution, and A_S_ is the absorbance after adding the sample solution. The IC_50_ value was processed by GraphPad Prism 8.

## Results

### Fragmentation Mechanisms of *Seco*-Sativene Sesquiterpenoids (1–8)

**SCHEME 1:**
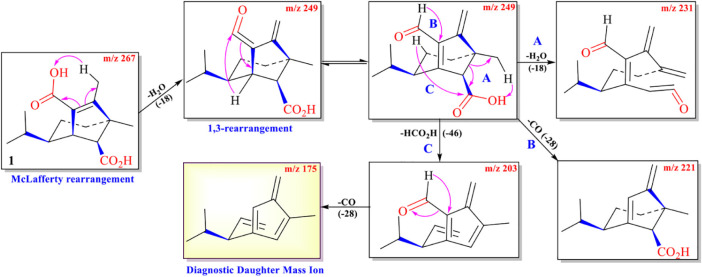
Possible fragmentation pathway of **1**.

The protonated parent ion *m*/*z* 267, [M+H]^+^ of cochliobolin A (**1**) was not observed in the UPLC-Q-TOF-MS/MS spectra, and it might be that this ion could easily lose one molecule of H_2_O to produce a daughter ion at *m*/*z* 249 [M+1-H_2_O]^+^. The abundance of ion *m*/*z* 249 was the highest in the profile. Thus, the precursor protonic molecular ion [M+1-H_2_O]^+^ of **1** was selected for analysis in the UPLC-Q-TOF-MS/MS spectrum. The high-resolution mass and fragment ions together with the elemental constituents of cochliobolin A (**1**) were listed in Table 2. The fragmentation routes according to ESI-Q-TOF-MS/MS analysis were depicted in Scheme 1, in which typical neutral losses, McLafferty rearrangement, and 1,3-rearrangement were the main fragmentation patterns for the parent ion *m*/*z* 267 [M+1]+ (Liang et al., 2018). The daughter ion (*m*/*z* 249) was formed from the parent ion (*m*/*z* 267) through the McLafferty rearrangement and 1,3 rearrangement by loss of one molecule of H_2_O (−18). Then, the daughter ion (*m*/*z* 221) was produced from the precursor ion (*m*/*z* 249) through neutral loss of one molecule of CO (−28). The daughter ion (*m*/*z* 231) was yielded from the precursor ion (*m*/*z* 249) through the McLafferty rearrangement by neutral loss of one molecule of H_2_O (−18). The diagnostic daughter ion (*m*/*z* 175) might have originated from the precursor ion (*m*/*z* 249) by loss of one molecule of HCO_2_H (−46) (*m*/*z* 203) and one molecule of CO (−28) (*m*/*z* 175) through the McLafferty rearrangement and neutral loss (Scheme 1 and Supplementary Figure 1).

**TABLE 2:** Elemental constituents of major product ions from [M+Na]^+^ for compound **1** (subtype A).

Fragment ion	Formula	Calculated	Observed	Error (PPM)
[M+Na]^+^	C_15_H_22_O_4_Na	289.1416	289.1412	−1.4
[M+H-H_2_O]^+^	C_15_H_21_O_3_	249.1491	249.1499	+3.2
[M+H-2H_2_O]^+^	C_15_H_19_O_2_	231.1385	231.1378	−3.0
[M+H-H_2_O-CO]^+^	C_14_H_21_O_2_	221.1542	221.1537	−2.3
[M+H-H_2_O-HCO_2_H]^+^	C_14_H_19_O	203.1436	203.1429	−3.4
[M+H-H_2_O-HCO_2_H-CO]^+^	C_13_H_19_	175.1487	175.1479	−4.6

**SCHEME 2:**
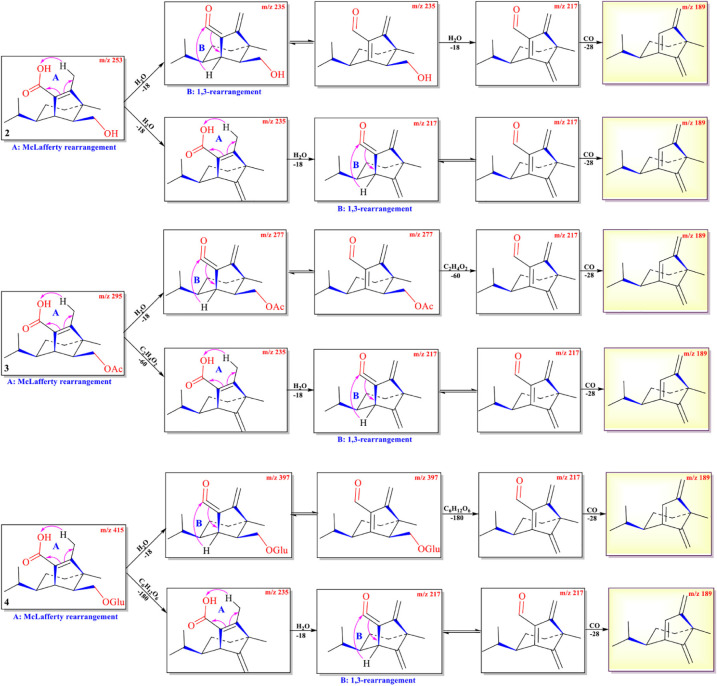
Possible fragmentation pathways of **2**–**4**.

The protonated parent ion *m*/*z* 253, [M+H]^+^ of compound **2** was observed in the mass profile with a relatively low abundance, which easily lost one molecule of H_2_O (−18) to produce the intermediate ion *m*/*z* 235 through the McLafferty rearrangement and 1,3-rearrangement or neutral loss of one molecule of H_2_O (–18) with H-13. Then, the intermediate ion *m*/*z* 235 successively lost one molecule of H_2_O (–18) and one molecule of CO (–28) by neutral loss to form the key diagnostic daughter ion (*m*/*z* 189). The high-resolution mass and fragment ions together with the elemental constituents of compound **2** were listed in Table 3. Compounds **3** and **4** had similar fragmentation pathways to that of compound **2** (Scheme 2, Supplementary Figures 2–4, and Supplementary Tables 1, 2).

**TABLE 3:** Elemental constituents of major product ions from [M+Na]^+^ for compound **2** (subtype B).

Fragment ion	Formula	Calculated	Observed	Error (PPM)
[M+Na]^+^	C_15_H_24_O_3_Na	275.1623	275.1618	−1.8
[M+H]^+^	C_15_H_25_O_3_	253.1804	253.1797	−2.8
[M+H-H_2_O]^+^	C_15_H_23_O_2_	235.1698	235.1703	+2.1
[M+H-2H_2_O]^+^	C_15_H_21_O	217.1592	217.1582	−4.6
[M+H-2H_2_O-CO]^+^	C_14_H_21_	189.1643	189.1631	−6.3

**SCHEME 3:**
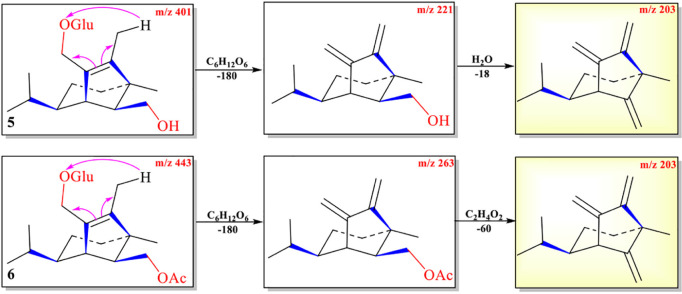
Possible fragmentation pathways of **5** and **6**.

The protonated parent ions of **5** and **6** were *m*/*z* 401 [M+H]^+^ and *m*/*z* 443 [M+H]^+^, respectively, in the UPLC-Q-TOF-MS/MS spectra. These two ions lost one molecule of glucose (–180) by the McLafferty rearrangement to form the intermediate ions *m*/*z* 221 and *m*/*z* 263, respectively, which yielded the same diagnostic ion *m*/*z* 203 through neutral loss of one molecule of H_2_O (−18) and one molecule of CH_3_COOH (−60) (Scheme 3 and Supplementary Figures 5, 6). The high-resolution mass and fragment ions together with the elemental constituents of compounds **5** and **6** were listed in Table 4 and Supplementary Table 3.

**TABLE 4:** Elemental constituents of major product ions from [M+Na]^+^ for compound **5** (subtype C).

Fragment ion	Formula	Calculated	Observed	Error (PPM)
[M+Na]^+^	C_21_H_36_O_7_Na	423.2359	423.2361	+0.5
[M+H]^+^	C_21_H_37_O_7_	401.2539	401.2535	−1.0
[M+H-C_6_H_12_O_6_]^+^	C_15_H_25_O	221.1905	221.1901	−1.8
[M+H-C_6_H_12_O_6_ -H_2_O]^+^	C_15_H_23_	203.1800	203.1790	−4.9

**SCHEME 4:**
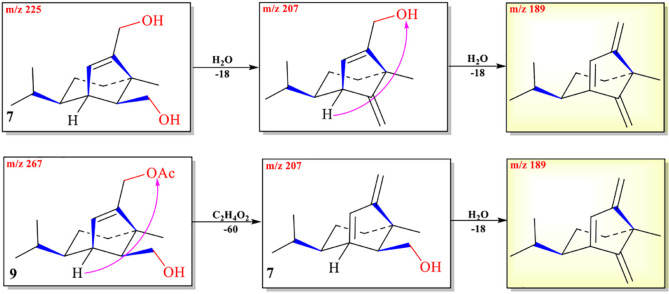
Possible fragmentation pathways of **7** and **9**.

The abundance of protonated parent ion of **7** *m*/*z* 225 [M+H]^+^ was relatively low in the UPLC-Q-TOF-MS/MS spectra. It might be the neutral loss of one molecule of H_2_O (−18) and one molecule of CH_3_CO_2_H (−60) to form an intermediate ion *m*/*z* 207. The abundance of the ion (*m*/*z* 207) was the highest in the mass profile. The diagnostic ion (*m/z* 189) was formed from ion *m*/*z* 207 by loss of one molecule of H_2_O (−18) through the McLafferty rearrangement (Scheme 4 and Supplementary Figure 7). The high-resolution mass and fragment ions together with the elemental constituents of compound **7** are listed in Table 5.

**TABLE 5:** Elemental constituents of major product ions from [M+Na]^+^ for compound **7** (subtype D).

Fragment ion	Formula	Calculated	Observed	Error (PPM)
[M+Na]^+^	C_14_H_24_O_2_Na	247.1674	247.1668	−2.4
[M+H]^+^	C_14_H_25_O_2_	225.1855	225.1843	−5.3
[M+H-H_2_O]^+^	C_14_H_23_O	207.1749	207.1754	+2.4
[M+H-2H_2_O]^+^	C_14_H_21_	189.1643	189.1640	−1.6

**SCHEME 5:**
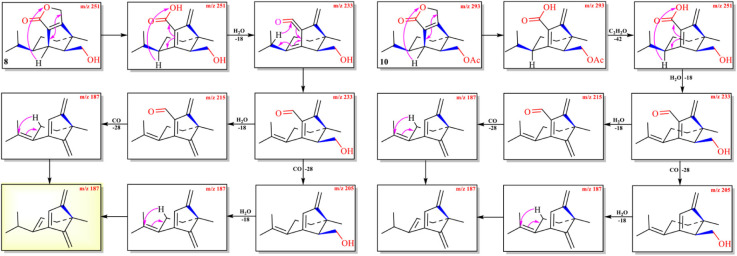
Possible fragmentation pathways of **8** and **10**.

The protonated parent ion of **8** was *m*/*z* 251 [M+H]^+^ in the UPLC-Q-TOF-MS/MS spectra with the highest abundance. The McLafferty rearrangement produced the intermediate ion *m*/*z* 233 from *m*/*z* 251 by loss of one molecule of H_2_O (−18). Successive neutral losses of one molecule of H_2_O (−18) and one molecule of CO (−28) or vice versa yielded the diagnostic daughter ion (*m*/*z* 187). The detailed MS analysis is shown in Scheme 5 and Supplementary Figure 8. The high-resolution mass and fragment ions together with the elemental constituents of compound **8** are listed in Table 6.

**TABLE 6:** Elemental constituents of major product ions from [M+Na]^+^ for compound **8** (subtype E).

Fragment ion	Formula	Calculated	Observed	Error (PPM)
[M+Na]^+^	C_15_H_22_O_3_Na	273.1467	273.1461	−2.2
[M+H]^+^	C_15_H_23_O_3_	251.1647	251.1664	+6.8
[M+H-H_2_O]^+^	C_15_H_21_O_2_	233.1542	233.1536	−2.6
[M+H-2H_2_O]^+^	C_15_H_19_O	215.1436	215.1426	−4.6
[M+H-H_2_O-CO]^+^	C_14_H_21_O	205.1592	205.1585	−3.4
[M+H-2H_2_O-CO]^+^	C_14_H_19_	187.1487	187.1478	−4.8

With the UPLC-Q-TOF-MS/MS fragmentation mechanisms of **1**–**8** in hand, it implied that each subtype *seco*-sativene sesquiterpenoids had a diagnostic daughter ion in the MS profile (subtype **A** → *m*/*z* 175; subtypes **B**/**D** → *m*/*z* 189; subtype **C** → *m*/*z* 203; subtype **E** → *m*/*z* 187). Though both subtypes **B** and **D** had the same diagnostic daughter ion *m*/*z* 189, the last cleavage in the sub-type **B** was the neutral loss of one molecule of CO (−28), whereas the neutral loss of one molecule of H_2_O (−18) was the last cleavage in subtype **D**, which differentiated these two subtypes **B** and **D**. Thus, it could give the possible subtype of *seco*-sativene sesquiterpenoids based on the diagnostic daughter ion from the corresponding ESI-Q-TOF-MS/MS data.

Then, the crude extract of the ethyl acetate fraction of the plant pathogen *Bipolaris sorokiniana* was then analyzed by UPLC-Q-TOF-MS/MS (Supplementary Figure 11). There were some peaks (compound **8**: *m*/*z* 251.1664, *t*_R_ = 3.53 min, λ_max_ = 217 nm; compound **7**: *m*/*z* 225.1843, *t*_R_ = 3.56 min, λ_max_ = 206 nm; compound **2**: *m*/*z* 253.1797, *t*_R_ = 4.86 min, λ_max_ = 244 nm; compound **1**: *m*/*z* 249.1548, *t*_R_ = 4.93 min, λ_max_ = 248 nm; compound **3**: *m*/*z* 295.1900, *t*_R_ = 8.24 min, λ_max_ = 243 nm) in the TOF MS profiles possessing typical fragment ions including *m*/*z* 175, 187, 189, 203, 205, 207, 215, 217, 231, 233, and 235. However, there are other unidentified compounds (251.1639, *t*_R_ = 2.85 min; 353.0418, *t*_R_ = 5.78 min; 237.1855, *t*_R_ = 5.88 min; 293.1748, *t*_R_ = 7.03 min; 207.1744, *t*_R_ = 7.26 min; 235.1693, *t*_R_ = 7.86 min; 235.1693, *t*_R_ = 8.68 min; 235.1692, *t*_R_ = 8.85 min) that had similar cleavage fragments as compounds **1**–**8**, indicating that many known and undescribed analogues existed in the extract.

Compounds **1** (*t*_R_ = 4.93 min) and **2** (*t*_R_ = 4.86 min) as the main constituents were isolated from the plant pathogen *Bipolaris sorokiniana* crude extract.

**FIGURE 2:**
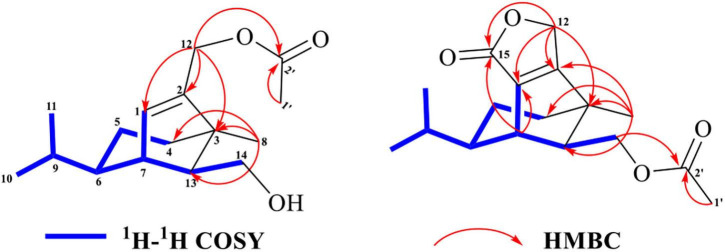
Key 2D-NMR correlations of **9** and **10**.

Two ions (*t*_R_ = 7.23, 7.01 min) at *m*/*z* 267 [M+1]^+^ (**9**) and *m*/*z* 293 [M+1]^+^ (**10**) were detected in the crude extract, and their molecular weights were determined to be C_16_H_26_O_3_ and C_17_H_24_O_4_, respectively (Supplementary Figures 9, 10). The diagnostic ions of **9** and **10** were *m*/*z* 189/187 and λ_max_ = 234/215 nm, respectively, implying that structures of **9** and **10** possessed the same subtypes as **7** and **8**. The UPLC-Q-TOF-MS/MS fragmentation pathways of these two compounds were nearly the same as those of **7** and **8**, except for an additional acetyl group. To confirm this hypothesis, **9** and **10** were isolated from the extract and elucidated by IR, NMR, and HR-ESI-MS spectra. The IR absorption bands at 3,420 cm^–1^ showed the hydroxyl group in **9**, and 2,931 and 1,747 cm^–1^ revealed the presence of alkyl and ester moieties, respectively, which were also present in that of **10**. The ^1^H-NMR spectrum revealed the similarity of **9**/**10** with **7**/**8**, except that the chemical shift values of –CH_2_-12 of **9** and –CH_2_-14 of **10** were down-fielded in **9**/**10** (Osterhage et al., 2002) and an additional methyl signal was observed in the ^1^H-NMR spectrum of **9**/**10**. This implied that the additional acetyl group was connected at C-12 in **9** and C-14 in **10**. The key HMBC correlations from –CH_2_-12/CH_2_-14 and 1′-Me to C-2′ (δ_C_ 171.2 in **9**/**10**) supported the conclusion (Figure 2 and Supplementary Figures 12–29). Thus, the structures of **9** and **10** were determined, and their ESI-Q-TOF-MS/MS fragmentation pathways were consistent with those of **7**/**8** (Schemes 4, 5 and Supplementary Tables 4, 5).

### Antioxidant Activity

The antioxidant activities of compounds **1** and **2** were evaluated by the DPPH and ABTS^+^ free radical scavenging test, and the results were presented as IC_50_ values. The results demonstrated that compound **1** displayed moderate antioxidant activities with IC_50_ of 0.90 and 1.97 mM for DPPH and ABTS^+^ scavenging capacity, respectively. However, compound **2** did not show the obvious antioxidant activity. They were measured by comparing the scavenging ability of DPPH free radical and ABTS^+^ free radical with V_C_, a well-known potent antioxidant and free radical scavenger with IC_50_ of 0.14 and 0.42 mM for DPPH and ABTS^+^ scavenging capacity, respectively (Table 7).

**TABLE 7:** DPPH and ABTS^+^ scavenging capacity of compounds **1**, **2**, **9**, and **10**.

Compound	DPPH (IC_50_)	ABTS^+^ (IC_50_)
1	0.90 ± 0.17 mM	>1 mM
2	>1 mM	>1 mM
9	>1 mM	>1 mM
10	>1 mM	>1 mM
Vc	0.14 ± 0.05 mM	0.42 ± 0.30 mM

## Discussion

This report analyzed the fragmentation patterns of eight representative *seco*-sativene sesquiterpenoids (**1**–**8**) using UPLC-Q-TOF-MS/MS, and McLafferty rearrangement, 1,3-rearrangement, and neural loss (−18, −28) were the main fragmentation patterns. The results indicated that dehydration (−18) occurred easily in *seco*-sativenes with strong abundance of dehydration peak observed in **1**-**8**, and similar reports were found in other studies (McLafferty and Gohike, 1959; Alén, 1987; Nawamaki and Kuroyanagi, 1996; Sun et al., 2012; Giri et al., 2017; Qian et al., 2018). This may be due to an electron impact inducing fragmentations of alkene monocarboxylic acids to form an active OH ion, which combines an available methyl-hydrogen atom to one lost molecule of H_2_O (−18) through the McLafferty rearrangement in a six-membered system (Baldas et al., 1969; Alexander et al., 1972). The molecular ion peak [M+H]^+^ of **1**–**7** and **9** was not easily observed in the UPLC-Q-TOF-MS/MS profile, whereas [M+Na]^+^ and [M+H-H_2_O]^+^ abundances of these compounds were relatively strong. As there was a characteristic 40 Da difference between [M+Na]^+^ and [M+H-H_2_O]^+^, the molecular ion peak of **1**–**7** and **9** can be inferred. Compounds **8** and **10** possess a lactone ring at C-1 and C-2, which is different from **1**–**7** and **9**. The special group in **8** and **10** leads to their molecular ion peaks [M+H]^+^ easily being observed in MS profiles. This might be a key signal for differentiating subtype E from other subtypes. Although the McLafferty rearrangement together with 1,3-rearrangement in alkene monocarboxylic molecules to produce a base peak of dehydration has been reported, this was the first report in *seco*-sativene sesquiterpenoids mass analysis, which provided a base for the *seco*-sativenes structural elucidation.

Diagnostic daughter ions of five subtypes of *seco*-sativene sesquiterpenoids were provided base on UPLC-Q-TOF-MS/MS analysis in this report. Subtypes A, B/D, and E possessed diagnostic daughter ions at *m*/*z* 175, 189, and 203, respectively, whereas subtype C showed a characteristic daughter ion at *m*/*z* 187 in the UPLC-Q-TOF-MS/MS profiles. The main difference between subtypes B and D was that the last cleavage was the neutral loss of one molecule of CO (−28) in subtype B, not the neutral loss of one molecule of H_2_O (−18) in subtype D. Diagnostic ions provided signals for the different subtypes of *seco*-sativenes, and the rearrangement and neutral loss (H_2_O, CO, and HOAc) in the fragmentation patterns provided the possible groups on the structures of *seco*-sativene sesquiterpenoids. Thus, the structures of the *seco*-sativenes could be inferred by fragmentation patterns combined with the diagnostic ions and molecular formula based on the UPLC-Q-TOF-MS/MS profile. This report provides a reliable method for the structural analysis of *seco*-sativene sesquiterpenoids.

Compounds **1**, **2**, **9**, and **10** and other *seco*-sativenes are a class of phytotoxins. Compound **1** was previously isolated from *C. sativus* (teleomorph: *B. sorokiniana*) without phytotoxicity on corn leaves, but helminthosporal acid with an aldehyde group at C-1 (a carboxyl group at C-1 in **1**) possessed strong phytotoxic activity (Li et al., 2020a). Therefore, the aldehyde group in helminthosporal acid might be a potential active group for phytotoxicity. Compound **2** exhibited bidirectional regulation activities. On the one hand, it was the gibberellin-like plant growth regulator, which could promote the growth of plant roots and leaves at low concentrations (Qader et al., 2017; Li et al., 2020a). On the other hand, it showed phytotoxicity on corn leaves (Li et al., 2020a). Compounds **9** and **10** did not show obvious antioxidant activity in this report, and no more activities of **9** and **10** were tested due to limited amounts. Compared with **7** and **8**, **9** and **10** possess an extra acetyl group at 12-OH and 5-OH, respectively. Osterhage et al. reported that **7** showed inhibitory activity against tyrosine kinase p56, *Microbotryum violaceum*, *Eurotium repens*, and *Escherichia coli* (Osterhage et al., 2002) and that **8** showed antifungal activity against *M. violaceum* (Osterhage et al., 2002) and phytotoxicity on corn leaves (Li et al., 2020a). Several reports suggested that 5-CH_2_OH might be the potent active group of *seco*-sativenes sesquiterpenoids for their phytotoxicity (Nakajima et al., 1994; Li et al., 2020b). Therefore, **9** might possess phytotoxicity against barley seeds and corn leaves. But their structure–activity relationships (SARs) need to be further studied. At present, the reports on the activities of *seco*-sativene sesquiterpenoids mainly focused on phytotoxicity and growth-promoting effects with few reports about other activities (Li et al., 2020b). To further explore the medicinal value of *seco*-sativenes, the antioxidant activity of these compounds (**1**, **2**, **9**, and **10**) were studied in this report. Only then did compound **1** show moderate activity on DPPH scavenging capacity, which indicated that 13-COOH might be a possible active group, and further biological exploration should be needed in the future.

UPLC-Q-TOF-MS/MS spectrometry has evolved to be a mature and common technique, which is now widely used to analyze secondary metabolites from diverse biological resources. Most of researchers used this technology to identify (new/novel) metabolites or dereplicate in different crude extracts (Jin et al., 2018; Wang et al., 2020). Recently, a molecular networking technique based on (U)HPLC-MS/MS combined with different databases was used in the dereplication and targeting of new natural products from diverse biological resources (Watrous et al., 2012; Yang et al., 2013; Aksenov et al., 2017; Hou et al., 2019; Ramos et al., 2019; Rivera-Chaìvez et al., 2019; Shi et al., 2019; Wu et al., 2019; Chao et al., 2020; Zang et al., 2020; Lei et al., 2021; Lin et al., 2021). The molecular networking technique used the known or new compound as the “seed” to realize the visualization of analogues. In the network, MS data were collected from LC-MS and uploaded to the GNPS database for data processing to produce total molecular network profiles. Every node in the same network represented a compound possessing the same core skeleton. Known or new analogues can be quickly inferred according to molecular weight, molecular formula, and fragmentation patterns based on node analysis through searching different databases or house libraries. The targeted isolation of the seed analogues could be realized by searching the location of the “seed” (Klitgaard et al., 2015; Allard et al., 2016; Trautman and Crawford, 2016; Naman et al., 2017; Olivon et al., 2017a,b; Nothias et al., 2018). When a molecular network is combined with fragmentation patterns, the range of metabolites would be narrowed, and the precision of targeted-isolation-compounds would be improved (He et al., 2021). Thus, molecular networking based on the (U)HPLC-MS/MS technique would provide a more convenient approach for dereplication and targeting-isolation of new *seco*-sativene sesquiterpenoids in the future.

## Conclusion

Eight *seco*-sativene sesquiterpenoids (**1**–**8**) were analyzed using the UPLC-Q-TOF-MS/MS technique in positive mode, from which their possible mass fragmentation patterns were suggested, and neural loss, McLafferty rearrangement, and 1,3-rearrangement were the main clearage patterns. These eight *seco*-sativene sesquiterpenoids (**1**–**8**) were summarized to be five subtypes according to their structural features. Each subtype possessed a diagnostic daughter ion, which, in return, could contribute to the elucidation of *seco*-sativene sesquiterpenoids. Based on the fragmentation mechanism mentioned above, some analogues including two potentially new ones were detected. Two known (**1** and **2**) and two new analogues (**9** and **10**) were then isolated from the extract of the plant pathogen *Bipolaris sorokiniana*. Their structures were elucidated mainly by NMR spectra and supported based on their UPLC-Q-TOF-MS/MS analysis. The results demonstrated that diagnostic mass ions of *seco*-sativene sesquiterpenoids in the UPLC-Q-TOF-MS/MS profiles provided a convenient and high-performance approach for structural characterization and also support that UPLC-Q-TOF-MS/MS is a powerful and sensitive tool for dereplication and detection of new analogues in crude extracts. This study will pave the way for the structural analysis and targeting isolation of *seco*-sativene sesquiterpenoids in different fungal crude extracts.

## Data Availability Statement

The original contributions presented in the study are included in the article/Supplementary Material, further inquiries can be directed to the corresponding author/s.

## Author Contributions

Y-DW, JY, and GD: experiment and writing – review and editing. Y-DW, JY, Y-YL, X-MT, and QL: data collection. S-YY and S-BN: activity experiment. HD: resources. JY, GD, and L-PG: funding acquisition. GD and Y-DW: writing – original draft preparation. All authors have read and agreed to the published version of the manuscript.

## Conflict of Interest

The authors declare that the research was conducted in the absence of any commercial or financial relationships that could be construed as a potential conflict of interest.

## Publisher’s Note

All claims expressed in this article are solely those of the authors and do not necessarily represent those of their affiliated organizations, or those of the publisher, the editors and the reviewers. Any product that may be evaluated in this article, or claim that may be made by its manufacturer, is not guaranteed or endorsed by the publisher.
