# Supplementary material for: UPLC-Q-TOF-MS/MS Analysis of Seco-Sativene Sesquiterpenoids to Detect New and Bioactive Analogues From Plant Pathogen Bipolaris sorokiniana
Source: Front Microbiol. 2022 Mar 9;13:807014. doi: 10.3389/fmicb.2022.807014 (PMC8959811; doi:10.3389/fmicb.2022.807014)
Supplement: Supplementary file 1 [file Data_Sheet_1.docx]

***Supplementary Material***

**UPLC-Q-TOF-MS/MS Analysis of *Seco*-sativene Sesquiterpenoids to Detect New Analogues from Plant Pathogen** ***Bipolaris sorokiniana***

Yan-Duo Wang ^1,†^, Jian Yang ^2,†^, Qi Li ^1^, Yuan-Yuan Li ^1^, Xiang-Mei Tan ^1^, Si-Yang Yao ^3^, Shu-Bin Niu ^3^, Hui Deng ^4^, Lan-Ping Guo ^2,*^, Gang Ding ^1,*^

^1^ Key Laboratory of Bioactive Substances and Resources Utilization of Chinese Herbal Medicine, Ministry of Education, Institute of Medicinal Plant Development, Chinese Academy of Medical Sciences and Peking Union Medical College, Beijing 100193, People’s Republic of China.; Tongguyin@163.com

^2^ State Key Laboratory Breeding Base of Dao-di Herbs, National Resource Center for Chinese Materia Medica, China Academy of Chinese Medical Sciences, Beijing 100700, People’s Republic of China.; Yangchem2012@163.com.

^3^ Department of Pharmacy, Beijing City University, Beijing 100083, People’s Republic of China.; niushubin0704@163.com

^4^ Key Laboratory of Microbial Resources, Ministry of Agriculture and Rural Affairs; Institute of Agricultural Resources and Regional Planning, Chinese Academy of Agricultural Sciences, Beijing 100081, People’s Republic of China.; denghui02@caas.cn

*****Correspondence: [dgfyhchina@163.com](mailto:dgfyhchina@163.com); [gding@implad.ac.cn](mailto:gding@implad.ac.cn), [glp01@126.com](mailto:glp01@126.com)

^†^ These authors have contributed equally to this work and share the first authorship.

**Table of Contents**

[Fig. S1 UPLC-Q-TOF-MS/MS spectra of compound (**1**) 3](#_Toc88767084)

[Fig. S2 UPLC-Q-TOF-MS/MS spectra of compound (**2**) 3](#_Toc88767085)

[Fig. S3 UPLC-Q-TOF-MS/MS spectra of compound (**3**) 3](#_Toc88767086)

[Fig. S4 UPLC-Q-TOF-MS/MS spectra of compound (**4**) 4](#_Toc88767087)

[Fig. S5 UPLC-Q-TOF-MS/MS spectra of compound (**5**) 4](#_Toc88767088)

[Fig. S6 UPLC-Q-TOF-MS/MS spectra of compound (**6**) 5](#_Toc88767089)

[Fig. S7 UPLC-Q-TOF-MS/MS spectra of compound (**7**) 5](#_Toc88767090)

[Fig. S8 UPLC-Q-TOF-MS/MS spectra of compound (**8**) 6](#_Toc88767091)

[Fig. S9 UPLC-Q-TOF-MS/MS spectra of compound (**9**) 6](#_Toc88767092)

[Fig. S10 UPLC-Q-TOF-MS/MS spectra of compound (**10**) 7](#_Toc88767093)

[Fig. S11 UPLC-Q-TOF-MS/MS profiles (positive mode) of the crude extract. 7](#_Toc88767094)

[Fig. S12 ^1^H NMR spectrum (500 MHz) of compound (**9**) in CDCl_3_. 8](#_Toc88767095)

[Fig. S13 ^13^C NMR spectrum (125 MHz) of compound (**9**) in CDCl_3_. 8](#_Toc88767096)

[Fig. S13a ^13^C NMR spectrum (125 MHz) of compound (**9**) in CDCl_3_. 9](#_Toc88767097)

[Fig. S14 ^1^H-^1^H COSY spectrum (500 MHz) of compound (**9**) in CDCl_3_. 9](#_Toc88767098)

[Fig. S15 HSQC spectrum (500 MHz) of compound (**9**) in CDCl_3_. 10](#_Toc88767099)

[Fig. S16 HMBC spectrum (500 MHz) of compound (**9**) in CDCl_3_. 10](#_Toc88767100)

[Fig. S17 NOESY spectrum (500 MHz) of compound (**9**) in CDCl_3_. 11](#_Toc88767101)

[Fig. S18 HRESIMS spectrum of compound (**9**). 12](#_Toc88767102)

[Fig. S19 UV spectrum of compound (**9**). 12](#_Toc88767104)

[Fig. S20 IR spectrum of compound (**9**). 13](#_Toc88767105)

[Fig. S21 ^1^H NMR spectrum (500 MHz) of compound (**10**) in CDCl_3_. 13](#_Toc88767106)

[Fig. S22a ^13^C NMR spectrum (125 MHz) of compound (**10**) in CDCl_3_. 14](#_Toc88767107)

[Fig. S22b ^13^C NMR spectrum (125 MHz) of compound (**10**) in CDCl_3_. 14](#_Toc88767108)

[Fig. S23 ^1^H-^1^H COSY spectrum (500 MHz) of compound (**10**) in CDCl_3_. 15](#_Toc88767109)

[Fig. S24 HSQC spectrum (500 MHz) of compound (**10**) in CDCl_3_. 15](#_Toc88767110)

[Fig. S25 HMBC spectrum (500 MHz) of compound (**10**) in CDCl_3_. 16](#_Toc88767111)

[Fig. S26 NOESY spectrum (500 MHz) of compound (**10**) in CDCl_3_. 16](#_Toc88767112)

[Fig. S27 HRESIMS spectrum of compound (**10**). 17](#_Toc88767113)

[Fig. S28 UV spectrum of compound (**10**). 17](#_Toc88767115)

[Fig. S29 IR spectrum of compound (**10**). 18](#_Toc88767116)

[Table. S1 Elemental constituents of major product ions from [M+Na]^+^ for compound (**3**) 18](#_Toc88767117)

[Table. S2 Elemental constituents of major product ions from [M+Na]^+^ for compound (**4**) 18](#_Toc88767118)

[Table. S3 Elemental constituents of major product ions from [M+Na]^+^ for compound (**6**) 19](#_Toc88767119)

[Table. S4 Elemental constituents of major product ions from [M+Na]^+^ for compound (**9**) 19](#_Toc88767120)

[Table. S5 Elemental constituents of major product ions from [M+Na]^+^ for compound (**10**) 19](#_Toc88767121)

# Fig. S1 UPLC-Q-TOF-MS/MS spectra of compound (**1**)

# Fig. S2 UPLC-Q-TOF-MS/MS spectra of compound (**2**)

# Fig. S3 UPLC-Q-TOF-MS/MS spectra of compound (**3**)

# Fig. S4 UPLC-Q-TOF-MS/MS spectra of compound (**4**)

# Fig. S5 UPLC-Q-TOF-MS/MS spectra of compound (**5**)

# Fig. S6 UPLC-Q-TOF-MS/MS spectra of compound (**6**)

# Fig. S7 UPLC-Q-TOF-MS/MS spectra of compound (**7**)

# Fig. S8 UPLC-Q-TOF-MS/MS spectra of compound (**8**)

# Fig. S9 UPLC-Q-TOF-MS/MS spectra of compound (**9**)

# Fig. S10 UPLC-Q-TOF-MS/MS spectra of compound (**10**)

# Fig. S11 UPLC-Q-TOF-MS/MS profiles (positive mode) of the crude extract of *Bipolaris sorokiniana*.

*Known compounds in the crude extract of *B. sorokiniana* were marked in pink, and unidentified compounds were marked in green.

# Fig. S12 ^1^H NMR spectrum (500 MHz) of compound (**9**) in CDCl_3_.

# Fig. S13 ^13^C NMR spectrum (125 MHz) of compound (**9**) in CDCl_3_.

# Fig. S13a ^13^C NMR spectrum (125 MHz) of compound (**9**) in CDCl_3_.

# Fig. S14 ^1^H-^1^H COSY spectrum (500 MHz) of compound (**9**) in CDCl_3_.

# Fig. S15 HSQC spectrum (500 MHz) of compound (**9**) in CDCl_3_.

# Fig. S16 HMBC spectrum (500 MHz) of compound (**9**) in CDCl_3_.

# Fig. S17 NOESY spectrum (500 MHz) of compound (**9**) in CDCl_3_.


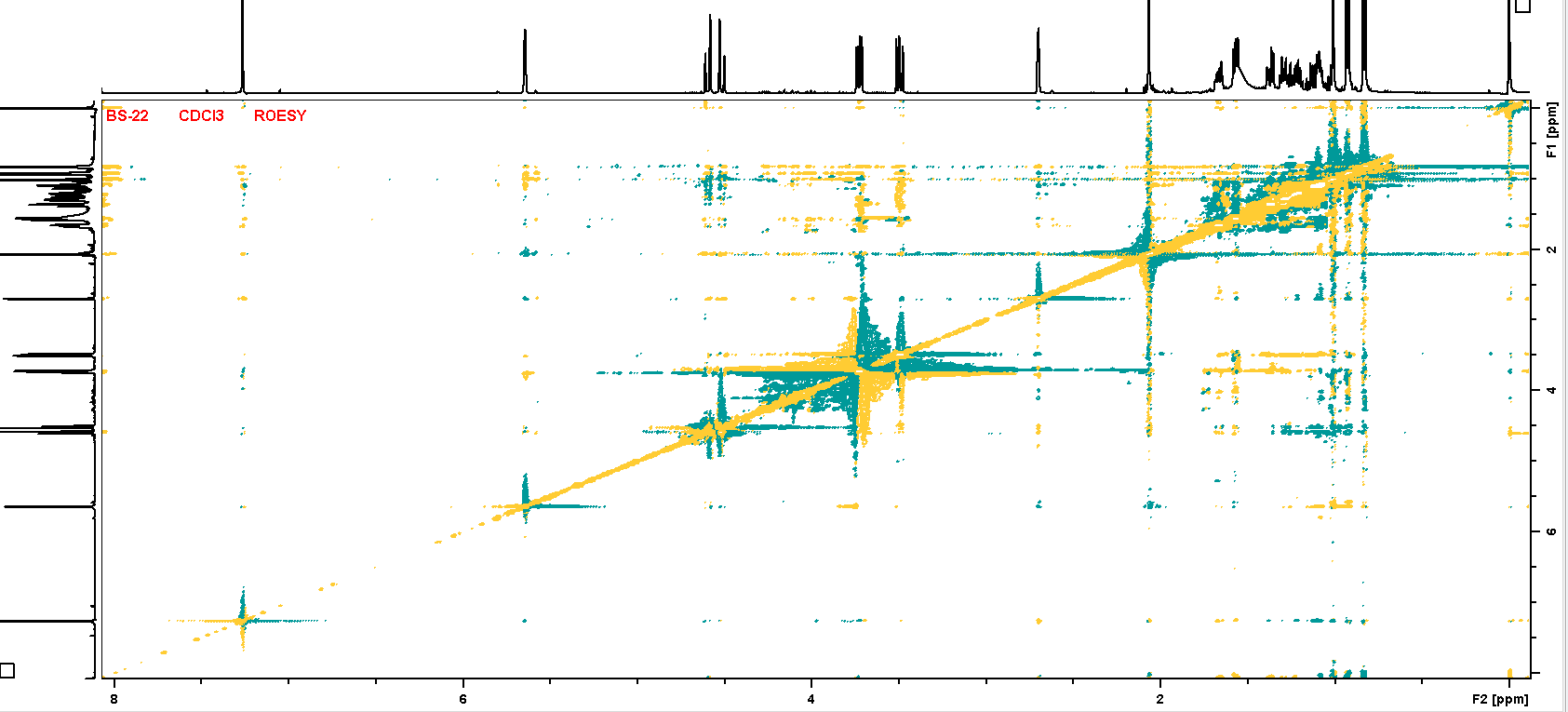


# Fig. S18 HRESIMS spectrum of compound (**9**).

# Fig. S19 UV spectrum of compound (**9**).

# Fig. S20 IR spectrum of compound (**9**).


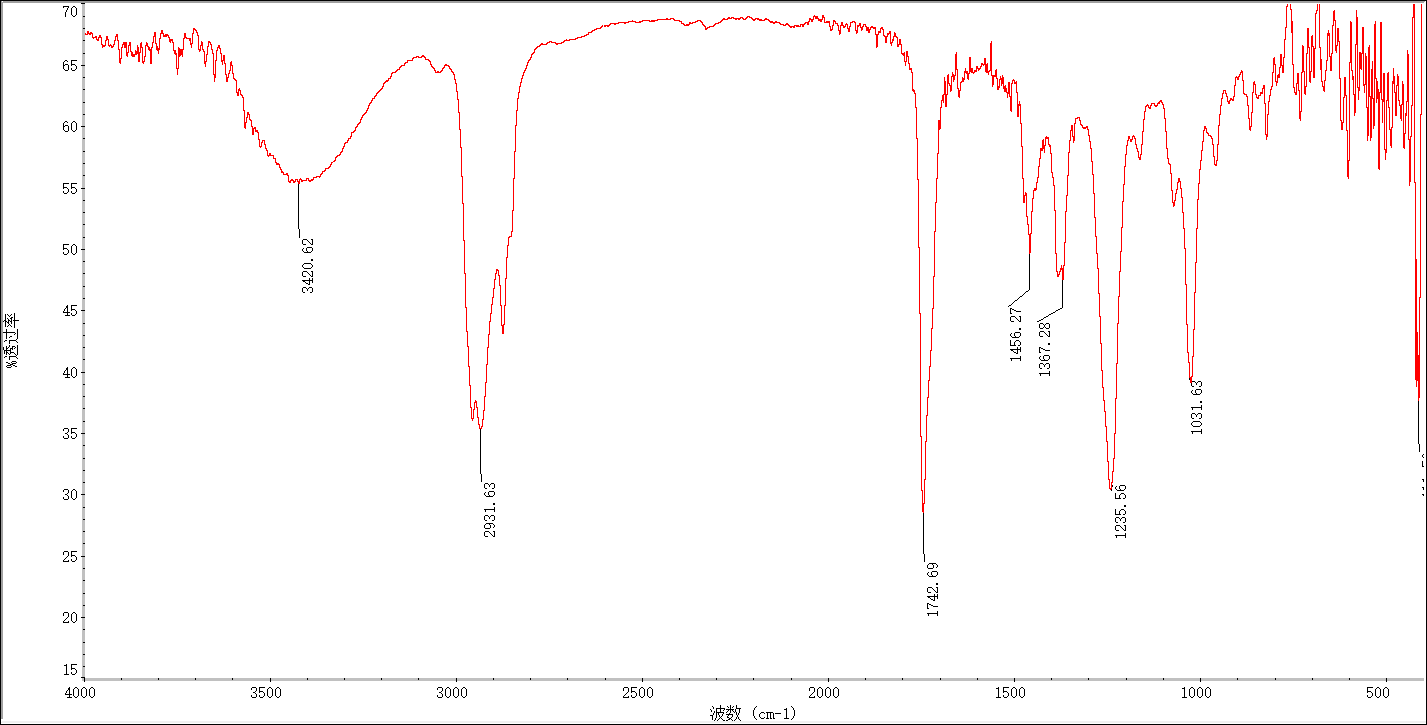


# Fig. S21 ^1^H NMR spectrum (500 MHz) of compound (**10**) in CDCl_3_.

# Fig. S22a ^13^C NMR spectrum (125 MHz) of compound (**10**) in CDCl_3_.

# Fig. S22b ^13^C NMR spectrum (125 MHz) of compound (**10**) in CDCl_3_.

# Fig. S23 ^1^H-^1^H COSY spectrum (500 MHz) of compound (**10**) in CDCl_3_.

# Fig. S24 HSQC spectrum (500 MHz) of compound (**10**) in CDCl_3_.

# Fig. S25 HMBC spectrum (500 MHz) of compound (**10**) in CDCl_3_.

# Fig. S26 NOESY spectrum (500 MHz) of compound (**10**) in CDCl_3_.


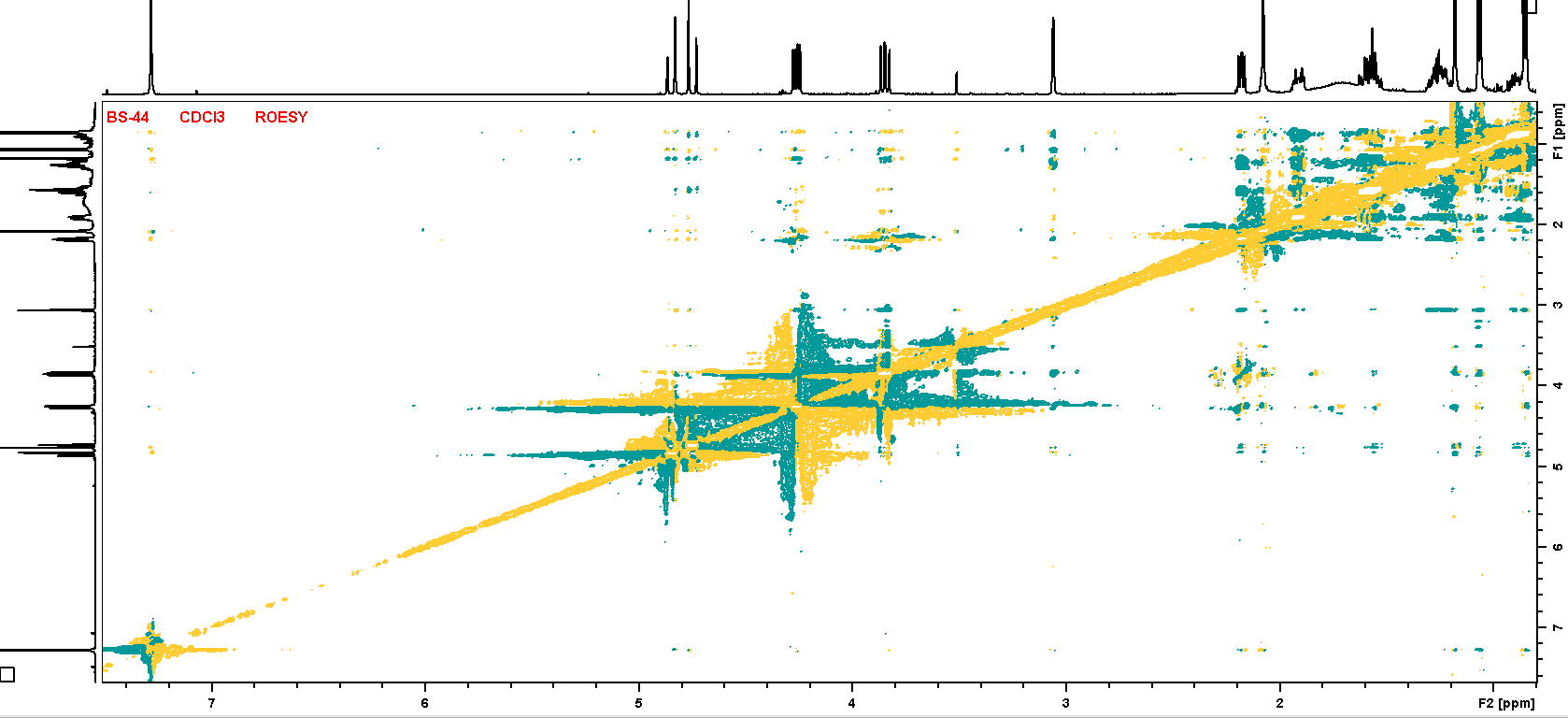


# Fig. S27 HRESIMS spectrum of compound (**10**).

# Fig. S28 UV spectrum of compound (**10**).

# Fig. S29 IR spectrum of compound (**10**).


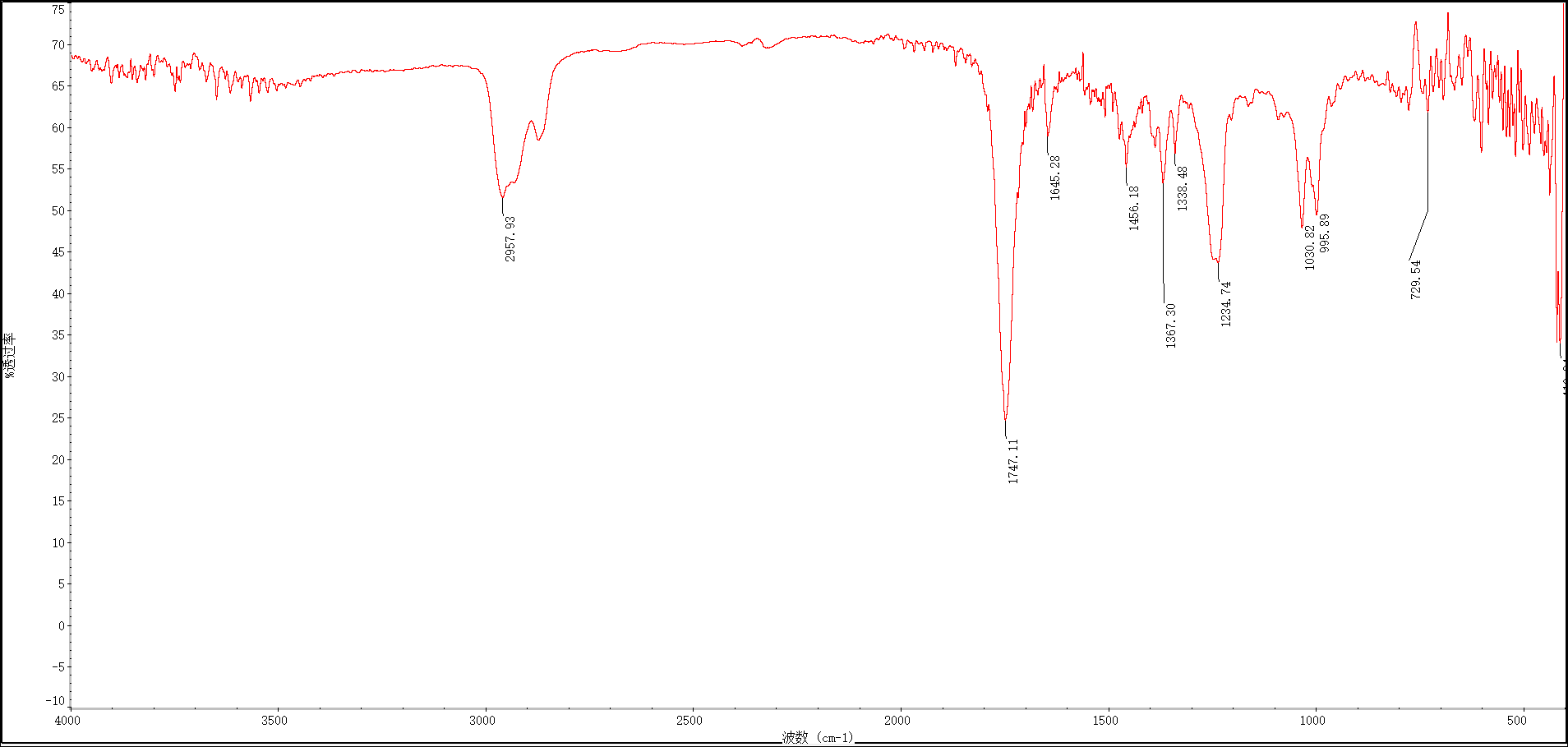


# Table. S1 Elemental constituents of major product ions from [M+Na]^+^ for compound (**3**)

| Fragment ion | Formula | Calculated | Observed | Error (PPM) |
| --- | --- | --- | --- | --- |
| [M+Na ]^+^ | C_17_H_26_O_4_Na | 317.1729 | 317.1720 | -2.8 |
| [M+H ]^+^ | C_17_H_27_O_4_ | 295.1909 | 295.1900 | -3.0 |
| [M+H-H_2_O]^+^ | C_17_H_25_O_3_ | 277.1804 | 277.1819 | +5.4 |
| [M+H -C_2_H_4_O_2_]^+^ | C_15_H_23_O_2_ | 235.1698 | 235.1689 | -3.8 |
| [M+H-H_2_O-C_2_H_4_O_2_]^+^ | C_15_H_21_O | 217.1592 | 217.1584 | -3.7 |
| [M+H-H_2_O-C_2_H_4_O_2_-CO]^+^ | C_14_H_21_ | 189.1643 | 189.1635 | -4.2 |

# Table. S2 Elemental constituents of major product ions from [M+Na]^+^ for compound (**4**)

| Fragment ion | Formula | Calculated | Observed | Error (PPM) |
| --- | --- | --- | --- | --- |
| [M+Na ]^+^ | C_21_H_34_O_8_Na | 437.2151 | 437.2149 | -0.5 |
| [M+H ]^+^ | C_21_H_35_O_8_ | 415.2332 | 415.2332 | 0.0 |
| [M+H-H_2_O]^+^ | C_21_H_33_O_7_ | 397.2226 | 397.2227 | +0.3 |
| [M+H-C_6_H_12_O_6_]^+^ | C_15_H_23_O_2_ | 235.1698 | 235.1702 | +1.7 |
| [M+H-H_2_O-C_6_H_12_O_6_]^+^ | C_15_H_21_O | 217.1592 | 217.1599 | +3.2 |
| [M+H-H_2_O-C_6_H_12_O_6_-CO]^+^ | C_14_H_21_ | 189.1643 | 189.1647 | +2.1 |

# Table. S3 Elemental constituents of major product ions from [M+Na]^+^ for compound (**6**)

| Fragment ion | Formula | Calculated | Observed | Error (PPM) |
| --- | --- | --- | --- | --- |
| [M+Na]^+^ | C_23_H_38_O_8_Na | 465.2464 | 465.2458 | -1.3 |
| [M+H]^+^ (no obeserved) | C_23_H_39_O_8_ |  |  |  |
| [M+H-C_6_H_12_O_6_]^+^ | C_17_H_27_O_2_ | 263.2011 | 263.2018 | +2.7 |
| [M+H-C_6_H_12_O_6_ -C_2_H_4_O_3_]^+^ | C_15_H_23_ | 203.1800 | 203.1799 | -0.5 |

# Table. S4 Elemental constituents of major product ions from [M+Na]^+^ for compound (**9**)

| Fragment ion | Formula | Calculated | Observed | Error (PPM) |
| --- | --- | --- | --- | --- |
| [M+Na]^+^ | C_16_H_26_O_3_Na | 289.1780 | 289.1775 | -1.7 |
| [M+H]^+^ | C_16_H_27_O_3_ | 267.1960 | 267.1957 | -1.1 |
| [M+H-C_2_H_4_O_2_]^+^ | C_14_H_23_O | 207.1749 | 207.1751 | +1.0 |
| [M+H-C_2_H_4_O_2_-2H_2_O]^+^ | C_14_H_21_ | 189.1643 | 189.1638 | -2.6 |

# Table. S5 Elemental constituents of major product ions from [M+Na]^+^ for compound (**10**)

| Fragment ion | Formula | Calculated | Observed | Error (PPM) |
| --- | --- | --- | --- | --- |
| [M+Na]^+^ | C_17_H_24_O_4_Na | 315.1572 | 315.1565 | -2.2 |
| [M+H]^+^ | C_17_H_25_O_4_ | 293.1753 | 293.1753 | 0.0 |
| [M+H-C_2_H_2_O]^+^ | C_15_H_23_O_3_ | 251.1647 | 251.1640 | -2.8 |
| [M+H-C_2_H_2_O-H_2_O]^+^ | C_15_H_21_O_2_ | 233.1542 | 233.1537 | -2.1 |
| [M+H-C_2_H_2_O-2H_2_O]^+^ | C_15_H_19_O | 215.1436 | 215.1430 | -2.8 |
| [M+H-C_2_H_2_O-H_2_O-CO]^+^ | C_14_H_21_O | 205.1592 | 205.1584 | -3.9 |
| [M+H-C_2_H_2_O-2H_2_O-CO]^+^ | C_14_H_19_ | 187.1487 | 187.1480 | -3.7 |
